# A Reversible Cause of Cutaneous Rash in a Patient With Alcohol Consumption

**DOI:** 10.7759/cureus.35011

**Published:** 2023-02-15

**Authors:** Asim Haider, Hitesh Gurjar, Haider Ghazanfar, Himani Singh, Ayesha Siddiqa

**Affiliations:** 1 Internal Medicine, BronxCare Health System, New York, USA; 2 Radiology, Ivy Hospital, Nawanshahr, IND; 3 Infectious Diseases, NewYork-Presbyterian Queens Hospital, New York, USA

**Keywords:** alcohol consumption, vitamin c, skin manifestation, cutaneous rash, scurvy, chronic alcoholism

## Abstract

The most common cutaneous manifestations of alcoholism include urticarial reaction, flushing, porphyria cutanea tarda, psoriasis, rosacea, seborrheic dermatitis, and pruritus. Here, we present a case of a young male with a history of alcohol abuse who presented with non-blanching, petechial, and perifollicular macular rash secondary to vitamin C deficiency in view of poor oral intake. The rash improved significantly with vitamin C supplementation. Although rare in developed countries, clinicians should keep vitamin C deficiency as a differential diagnosis for skin rash in alcohol consumers.

## Introduction

Alcohol abuse is a major problem in the United States (US) and is associated with significant morbidity and mortality. A meta-analysis of six national surveys from 2000 to 2016 showed that the prevalence of alcohol use and binge drinking had increased by 0.30% and 0.72% per year, respectively [1]. According to the 2019 National Survey on Drug Use and Health (NSDUH), about 25.8% and 6.3% of people aged 18 years and older in the US reported binge alcohol drinking and heavy alcohol use, respectively [2].

The coronavirus disease 2019 (COVID-19) pandemic has worsened the alcohol abuse problem. A survey showed that alcohol consumption had increased by 14% in the pandemic compared to 2019 [3]. Another study reported an increase of 29% (p < 0.001) and 21% (p = 0.001) in average drinks per day and binge drinking during the pandemic, respectively [4]. Increased alcohol consumption during the COVID-19 pandemic is due to increased stress, increased alcohol availability, confinement, and boredom [5].

An increase in alcoholic consumption has directly led to an increase in the prevalence of alcohol-related diseases. Scurvy is a disease caused by severe vitamin C deficiency and is rarely seen in developed countries due to improved nutritional status [6]. We report a case of alcohol-induced scurvy in a 23-year-old male patient.

## Case presentation

The patient was a 23-year-old male who presented to the emergency department with the complaint of insect crawling sensation and generalized rash on his body for the past one day. He had a pruritic rash that started on the leg and rapidly spread throughout his body. The past medical history was significant for chronic alcohol abuse. He reported drinking 12 cans of beer per day for the last eight months. The previous alcohol intake was 24 hours before the presentation. He also reported severe nausea. He had no prior surgical history. His mother had diabetes mellitus. He denied using any new medications or herbal products. The patient had an intolerance to ibuprofen and penicillin.

**Figure 1:**
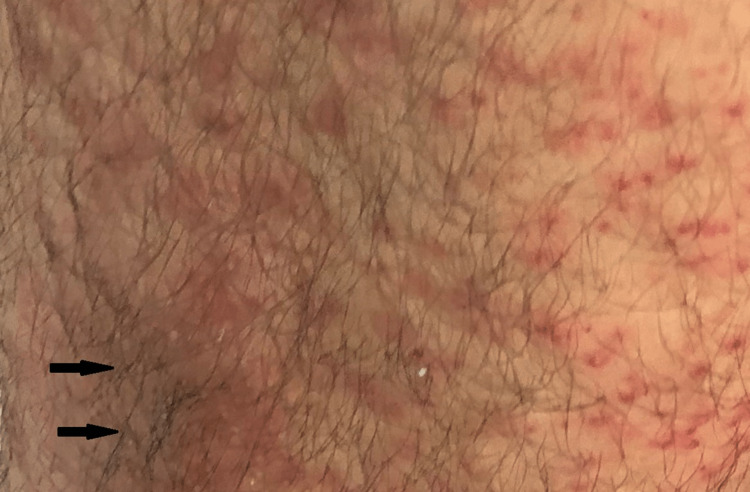
A magnified view of the left shin showing petechial, perifollicular macules with an erythematous base (black arrows)

**Figure 2:**
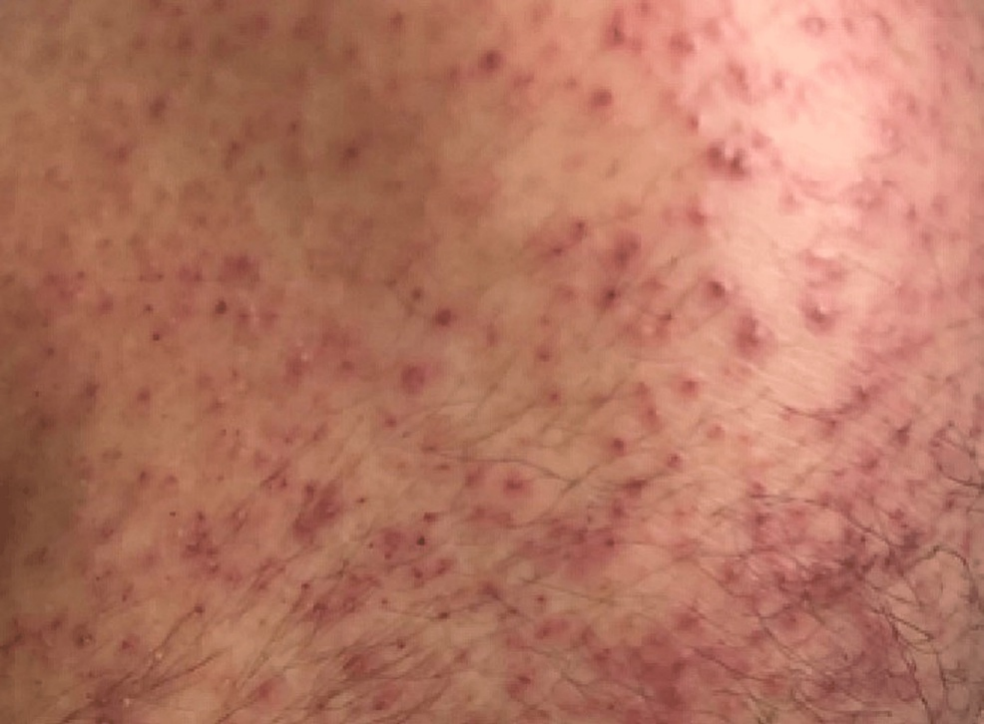
A magnified view of the right knee showing perifollicular rash

Upon presentation, he had a temperature of 99°F, a heart rate of 129 beats per minute, blood pressure of 149/89 mmHg, and oxygen saturation of 98% on room air. He had an unkempt appearance and was jittery, with mild tremors in his extremities. The Clinical Institute Withdrawal Assessment for Alcohol revised (CIWA-Ar) score was 12. He had perifollicular, non-blanching petechial macules with an erythematous base on the extremities and trunk, as seen in Figures 1, 2.

He had vesicular breathing sounds bilaterally and normal heart sounds. The abdominal and neurological examinations were unremarkable. The patient was admitted to the intensive care unit (ICU) for the management of alcohol withdrawal. He was started on intravenous fluids, multivitamins, and chlordiazepoxide 50 milligrams every eight hours. Because there was no history of exposure to any new drugs, antibiotics, or herbal products, the initial impression of the rash was vasculitis. The laboratory findings are summarized in Table 1.

**Table 1:** Summary of baseline laboratory findings

Parameter	Value	Reference range
Hemoglobin (gram per deciliter)	10.4	12.0-16.0
White blood cell count (x10^3^ per micro-liter)	8.9	4.8-10.8
Platelet (x10^3^ per micro-liter)	405	150-400
Sodium, serum (milliequivalents per liter)	134	135-145
Potassium, serum (milliequivalents per liter)	3.8	3.5-5.0
Blood urea nitrogen, serum (milligram liter)	5.0	6.0-20.0
Creatinine, serum (milligram per deciliter)	0.4	0.5-1.5
Alanine aminotransferase, serum ( unit per liter)	43	<5-40
Aspartate aminotransferase, serum ( unit per liter)	82	9-48
Blood glucose level, serum (milligram per deciliter)	77	70-120
Prothrombin time	13.4	9.9-13.3 seconds
Partial thromboplastin time	32.8	27.2-39.6 seconds

In view of the pigmented petechial rash, wide differentials were initially considered, including nutritional, leukocytoclastic vasculitic, and drug-induced. The causes can also be grouped into vessel wall related, such as amyloid or calcium deposit; surrounding connective tissue causes, such as senile purpura and scurvy; or vascular thrombotic causes, such as leukocytoclastic causes, including cryoglobulinemia. In view of the widespread rash, a dermatologist review was requested, and further workup was planned. He was worked up for autoimmune causes as summarized in Table 2. In view of the association of alcohol use with porphyria cutanea tarda and hepatitis C, the serology was sent, which turned out to be negative. The cryoglobulin levels were reported normal. Hyperglobulinemic purpura, which is closely associated with petechial rash, was ruled out by normal immunoglobulin levels. Along with this, the ascorbic acid deficiency was also thought of as one of the differentials, and vitamin C levels were sent. The patient was empirically started on 1 gram of ascorbic acid daily.

**Table 2:** Summary of laboratory parameters pertaining to differential diagnoses

Parameter	Value	Reference range
Antinuclear antibodies (ANA)	Negative	Negative
Anti-cyclic citrullinated antibody	<16	<20
Anti-mitochondrial antibody	Negative	Negative
Hepatitis C virus antibody	Non-reactive	Non-reactive
Human immunodeficiency virus RNA viral load	Target not detected	Target not detected
Antineutrophil cytoplasmic antibodies (ANCA)	<1.0	<1.0: no antibody detected; > or = 1.0: antibody detected
Sjogren’s antibody	<1.0	<1.0
Serum vitamin B1 (nmol/L)	689	78-185
Serum vitamin B12 (pg/mL)	1101	243-894
Serum vitamin D 25 OH (ng/mL)	9.1	30-100 ng/mL
Serum vitamin K (pg/ml)	283	130-1500
Autoantibodies to ribosomal P proteins	<1.0	<1.0
Anti-ribonucleoprotein (RNP) antibodies	<1.0	<1.0
Anti-Smith antibodies	<1.0	<1.0
Serum C3 complement (mg/dL)	113.0	90.0-150
Serum C4 complement (mg/dL)	17	16.0-47.0
Glomerular basement membrane antibody	<1.0	<1.0 AI: no antibody detected; > or = 1.0 AI: antibody detected
Anti-DNA antibody	1	<4
Hepatitis B surface antigen	Non-reactive	Non-reactive
Hepatitis B surface antibody	Non-reactive	Non-reactive
Rubeola immunoglobulin G antibody	172.00	>16.49 consistent with immunity
Cryoglobulin, qualitative	None detected	None detected
Measles immunoglobulin M antibody titer	<1:20	<1:20: antibody not detected; > or = 1:20: antibody detected
Serum immunoglobulin A (mg/dl)	251	47-310
Serum immunoglobulin G (mg/dl)	954	600-1640
Serum immunoglobulin M (mg/dl)	127	50-300
B2 glycoprotein 1 (U/ml)	<2	<20
Cardiolipin antibody (U/ml)	2.7	<20.0: antibody not detected; > or = 20.0: antibody detected
Lupus anticoagulant	27	<45 seconds

The rash started improving after one week, and the levels of vitamin C were reported as undetectable (<0.1 mg/dl; reference range: 0.2-2.1), thus confirming the diagnosis of scurvy. In some cases, when there is no response, other causes, such as sarcoidosis and Kaposi’s sarcoma, can also be considered, requiring skin biopsy for confirmation. The index patient refused skin biopsy as he started improving with ascorbic acid supplementation.

## Discussion

Scurvy is a disease caused by a deficiency of vitamin C leading to skin changes, perifollicular hemorrhages, capillary fragility, ecchymoses, gum bleeding, muscle pain, and bone fractures. Recent anthropologic studies based on bony changes have found that scurvy was also common in the ancient human population [7,8]. As humans lack gluconolactone oxidase, which synthesizes ascorbic acid, hence it is required as a supplement. It is a water-soluble vitamin widely available in fruits and the population eating a normal diet in developed countries rarely develops its deficiency. Alcohol consumers are especially at risk given their dietary habits and poor intake of nutritional supplements. Differential diagnosis of perifollicular petechial rash is tabulated in Table 3.

**Table 3:** Differential diagnosis of perifollicular petechial rash ANCA: antineutrophil cytoplasmic antibodies; APLA: antiphospholipid antibodies; TTP: thrombotic thrombocytopenia purpura; anti-CCP: anti-cyclic citrullinated peptide.

Diseases	Clinical features	Diagnostic features and workup
Scurvy	4H’s: hemorrhagic signs, hyperkeratosis of hair, hypochondriasis, hematological abnormalities; bony pains: subperiosteal hemorrhages; nails: splinter hemorrhages	Clinical history, skin findings, serum or leukocyte ascorbate levels
Leukocytoclastic vasculitis	Clues: neural, lung, or renal involvement. Examples: rheumatoid Arthritis, ANCA-associated vasculitis, cryoglobulinemia	ANCA, rheumatoid factor, anti-CCP, cryoglobulin levels
Pseudovasculitis	APLA, TTP, lymphoma, warfarin-induced skin necrosis, amyloid	Antiphospholipid antibodies, beta-2 glycoprotein, lupus anticoagulant, serum immunofixation, flow cytometry, ADAMTS-13
Infectious diseases	Rickettsial infections, hepatitis C infection	Rickettsial serology, hepatitis B and C serology
Other causes	Drugs, cocaine, chemotherapy	Careful history

Scurvy can cause a wide gamut of changes as vitamin C is required for the activation of a variety of copper and iron-containing enzymes. It is required in post-synthetic modifications of procollagen to collagen; hence its deficiency causes characteristic skin changes. It is also required in post-synthetic modification of protein C, which in turn hydrolyses activated factor V; this combined with the deficient collagen causes capillary fragility, perifollicular hemorrhages, and gum bleeding. In severe unrecognized cases, it can even cause hemarthroses and rarely cerebral hemorrhages [9]. It is also required for the formation of osteocalcin and its deficiency leads to bony changes and fractures causing characteristic radiologic signs such as the Frenkel line, Pelkan spur, Trummerfeld zone, or Wimberger ring sign [10]. It is also required in the synthesis of catecholamine (via a reduction reaction of copper-containing dopamine beta-hydroxylase enzyme) and complement C1q synthesis. Given these widespread changes, it is important to diagnose scurvy, which is characterized by typical morphological skin (perifollicular petechial hemorrhages) and hair follicle changes (corkscrew or swan neck shape) [7,9]. It is important to differentiate it from other diseases causing similar skin changes such as leukocytoclastic vasculitis, cryoglobulinemia, hyperglobulinemic purpura, and drug reactions [9,11,12]. Diagnoses can be confirmed by measuring serum ascorbic acid levels or leucocyte ascorbate levels. Replacing the ascorbate in dosages of 1-2 grams can correct the underlying deficiency. The perifollicular rash usually improves with one to two weeks of vitamin C supplementation while the corkscrew hair usually takes four weeks to regain normal appearance [6].

## Conclusions

The common causes of cutaneous rash in alcohol consumers include urticarial reaction, flushing, porphyria cutanea tarda, psoriasis, rosacea, seborrheic dermatitis, and pruritus. Vitamin C deficiency should be considered as a differential diagnosis for skin rash in a malnourished patient with a history of alcohol abuse. Vitamin C deficiency is uncommon in developed countries and is often overlooked.
